# Experiences of cervical screening and barriers to participation in the context of an organised programme: a systematic review and thematic synthesis

**DOI:** 10.1002/pon.4126

**Published:** 2016-04-12

**Authors:** Amanda J. Chorley, Laura A. V. Marlow, Alice S. Forster, Jessica B. Haddrell, Jo Waller

**Affiliations:** ^1^ Cancer Research UK Health Behaviour Research Centre, Department of Epidemiology and Public HealthUCLLondonUK

**Keywords:** Pap smear, attitudes, disparities, cancer, oncology

## Abstract

**Objective:**

As uptake of cervical screening continues to decline, this systematic review synthesises the qualitative literature on women's perceptions and experiences of cervical screening in the context of an organised call–recall programme, in order to understand the barriers to informed uptake.

**Methods:**

We searched nine databases for English language peer‐reviewed publications reporting on qualitative data from screening‐eligible women, exploring barriers to cervical screening in countries that offer a nationally organised call–recall programme. Evidence was integrated using thematic synthesis.

**Results:**

Thirty‐nine papers from the UK, Australia, Sweden and Korea were included. The majority of participants had attended screening at least once. Two broad themes were identified: (a) should I go for screening? and (b) screening is a big deal. In considering whether to attend, women discussed the personal relevance and value of screening. Women who had previously attended described how it was a big deal, physically and emotionally, and the varied threats that screening presents. Practical barriers affected whether women translated screening intentions into action.

**Conclusions:**

The variation in women's understanding and perceptions of cervical screening suggests that interventions tailored to decisional stage may be of value in increasing engagement with the invitation and uptake of screening in those who wish to take part. There is also a need for further research with women who have never attended screening, especially those who remain unaware or unengaged, as their perspectives are lacking in the existing literature. © 2016 The Authors. Psycho‐Oncology Published by John Wiley & Sons Ltd.

## Background

In recent decades, many countries have had great success in reducing cervical cancer incidence and mortality through offering population‐based screening programmes, using centralised registers of eligible women to generate invitations at regular intervals. For example, the national screening programme in the UK has averted an estimated 100 000 deaths from cervical cancer since its inception in 1988 1. Such systems remove the need for women to remember that they need screening and provide details of how to go about it. Many programmes also offer screening free at the point of delivery, reducing financial barriers.

Despite this, there are still many women invited to participate who do not attend according to recommendations, and in recent years, coverage of many programmes has been suboptimal. In the England in 2014–2015, age‐appropriate coverage^1^ was 73.5% 2, with coverage for women aged 25–29 years just 63.3%. Heterogeneity also exists in coverage by ethnic and socioeconomic group, with lower attendance in Black and Asian minority ethnic (BAME) groups 3 and women from socioeconomically deprived backgrounds 4, 5.

When considering programme coverage, it is important to remember that levels of attendance rely on individual women making the decision to attend and translating that decision into action. Therefore, research exploring the experiences and attitudes of the programme‐eligible population is important in informing policy and intervention design. A range of reasons for nonattendance have been identified using qualitative methods, including attitudinal factors such as embarrassment or fear of an abnormal result, as well as practical barriers such as clinic opening hours or availability of childcare. Despite the wealth of evidence available, the process of reading and interpreting original qualitative research can be arduous, and studies are not designed to be representative.

Previous reviews have drawn together evidence on barriers to and experiences of screening for specific population subgroups 6, 7, 8, 9, 10, 11, within a particular country 12, from specific theoretical viewpoints 13, or with a focus on a singular aspect of screening such as risk perceptions 14. However, no attempt has been made to synthesise the existing qualitative literature in a way that incorporates all women regardless of screening history or demographic characteristics, within a shared screening context across multiple countries. Synthesising qualitative research is a relatively new method that seeks to generate a novel interpretation of a phenomenon through the comparison and translation of concepts across studies, while maintaining rigorous and transparent standards of analysis 15, 16, 17, 18. This allows for the generation of new knowledge, which ‘goes beyond’ that in any original piece of research, and produces a coherent, convincing argument about the major attributes of the phenomenon of interest.

The aim of the current study was to carry out a synthesis of the available qualitative research on women's perceptions and experiences of screening, allowing the identification of important themes across contexts, increasing generalisability and creating practically useful information.

## Methods

This review was prospectively registered with PROSPERO (registration number: CRD42015017075) and reported according to PRISMA 19 and ENTREQ 20 guidelines.

### Search strategy

On 12 February 2015, we conducted a comprehensive search of published qualitative literature, using MEDLINE, PsycINFO, Embase, Social Policy and Practice, CINAHL Plus, ProQuest Social Science Journals, Anthrosource, POPLINE and Web of Science. Articles were included if they reported primary qualitative data on women's perception or experiences of cervical screening and were based in a country with a well‐established (10+ years) call–recall programme (Supporting Information 1). Articles also had to be available in English and have a publication date after the start of the organised cervical screening programme in the country in question. Search terms (Supporting Information 2) covered cervical screening (e.g. ‘smear test’), qualitative methodology (e.g. ‘focus groups’), barriers to screening (e.g. ‘perception’) and country of origin (e.g. ‘Australia’) and were linked using Boolean operators, with truncations and wildcards where appropriate. Additional articles were identified through the reference lists of included articles and excluded review articles, and forward citation searches.

### Data extraction

After the removal of duplicates, all articles were indexed in Microsoft Excel. A. C. and L. M. screened all titles and abstracts and, subsequently, the full text of remaining articles, to establish whether they met the inclusion criteria. At each stage, disagreement was resolved through discussion. Primary outcome data, defined as participant quotes and authors' interpretation of textual data, were extracted into a Microsoft Word document.

### Data synthesis

Articles were synthesised according to the principles of thematic synthesis 21, chosen because of its explicit focus on creating an end product, which is useful and accessible to researchers and policy makers and in intervention design. Initially, A. C., L. M. and J. W. independently carried out free coding of one third of the data, with codes generated inductively. The similarities and differences in identified codes were discussed and arranged into hierarchical groupings, which fully described emergent themes. A coding frame that represented concepts across all studies was developed and applied to the data by A. C. and L. M. using nvivo 10 (QSR International, Melbourne, Australia) 22, with refinements made after the coding of 10 articles. All text was coded, unless it met specific exclusion criteria (Supporting Information 3). Uncertainties regarding coding were resolved through discussion. Finally, A. C., L. M. and J. W. familiarised themselves with the content of each code, and higher order themes were developed through discussion.

### Quality assessment

The quality of included studies was assessed by A. C. and J. H. using the critical appraisal skills programme (CASP) qualitative checklist 23. Studies with a score of 0–4 were considered to be low quality, while scores of 5–9 were considered high quality. No study was excluded on the basis of quality.

## Results

Overall, 450 unique articles were identified. After screening of titles and abstracts, 103 articles remained, and the full texts were reviewed. From these, 32 articles met the eligibility criteria, with a further seven articles identified through reference lists and forward citation searches. Thirty‐nine articles were included in the final synthesis (Supporting Information 4). Only four articles had a CASP appraisal score of less than five (out of nine). As no theme relied on a single study, it is unlikely that inclusion of these studies substantially affected the results.

Included studies were published between 1988 and 2015 (see Table 1 for full characteristics). Over half were based in the UK (51.3%), with others coming from Australia (28.2%), Sweden (17.9%) and Korea (2.6%). Studies used interviews (51.3%), focus groups (30.8%) or a combination of the two (17.9%). Three studies (7.7%) also used open text responses from surveys or anonymous fax messages. Almost half of the articles (48.7%) focused on a specific subgroup of the population, mostly BAME women (*n* = 14). Most women across studies had attended cervical screening at least once. However, a number of studies included women who had never attended. Four studies (with women from BAME groups and deaf women) included some participants who were unaware of screening 24, 43, 49, 62. Despite their presence, the perspective of never attenders was far less explored. Because of this, the identified themes are predominantly shaped by the perceptions of women who had at least one experience of screening to draw upon.

**Table 1:** Characteristics of studies included in review

		Country	Eligible participants^a^ (*n*)	Age range (years)	Population	Study design	Analytic method	CASP score
Abdullahi *et al.* (2009)	24	UK	50	25–64	Somali‐born women	Focus groups + in‐depth interviews	Thematic analysis	8
Armstrong (2005)^b^	25	UK	35	20–64^c^	Nonspecific	Depth interviews	Inductive	6
Armstrong (2007)^b^	26	UK	35	20–64^c^	Nonspecific	In‐depth interviews	Inductive	5
Armstrong *et al.* (2012)^b^	27	UK	34	26–60	Nonspecific	Semistructured interviews	Constant comparative method	8
Blomberg *et al.* (2008)	28	Sweden	98	Not stated	Women actively declining cervical screening participation	Telephone interviews and fax messages	Interpretive description	8
Blomberg *et al.* (2011a)^d^	29	Sweden	138	30	Thirty‐year‐old women	Focus groups (face to face and Internet based) + one interview	Interpretive description	8
Blomberg *et al.* (2011b)	30	Sweden	38	29–32	Thirty‐year‐old women + convenience sample of three women aged 29–32 years	Focus groups + one interview	Inductive	8
Box (1998)	31	UK	17	Min. 16	BAME women	In‐depth interviews	Not stated	4
Broughton and Thomson (2000)	32	UK	52	20–64	Women with mild to moderate learning disabilities	Semistructured interviews	Thematic approach	8
Bush (2000)	33	UK	35	20–64	Nonspecific	Semistructured in‐depth interviews + qualitative comments from a survey	Not stated	7
Cadman *et al.* (2015)	34	UK	23	23–63	Hindu women	Focus groups	Framework analysis	8
Cadman *et al.* (2012)	35	UK	124	20–59	Women reporting a history of sexual abuse	Qualitative survey responses + one focus group	Content analysis	8
Chiu *et al.* (1999)	36	UK	27	Not stated	BAME women	Focus groups	Discursive strategy	7
Elkind *et al.* (1988)	37	UK	56	Not stated	Women noted as ‘did not attend’ in health authority records	Interviews	Not stated	5
Emami and Tishelman (2004)	38	Sweden	45	25–70^c^	Iranian immigrant women	Focus groups	Not stated	8
Forss *et al.* (2001)	39	Sweden	66 (with three transcripts excluded)	25–60	Cervical screening attenders	Unstructured interviews	Modified phenomenographic approach	9
Gregory and McKie (1991)	40	UK	72	20 to mid‐60s	Nonspecific	Focus groups	Not stated	4
Jackowska *et al.* (2012)	41	UK	52	20–55	Polish, Romanian and Slovakian women	Focus groups and interviews	Framework analysis	8
Jirojwong and Manderson (2001)	42	Australia	6	Not stated	Thai immigrant women	In‐depth interviews	Content analysis	4
Kwok *et al.* (2011)	43	Australia	18	28–66	Chinese‐Australian women	In‐depth interviews	Content analysis	7
Logan and McIlfatrick (2011)	44	UK	48	18–65^c^	Women living in socially deprived areas	Focus groups	Thematic content analysis	8
Manderson and Hoban (2006)	45	Australia	368^e^	Not stated	Indigenous women	Focus groups, in‐depth interviews and case histories	Thematic analysis	9
McKie (1995)	46	UK	72	18–73	White British, working‐class women	Focus groups	Not stated	6
Milburn and MacAskill (1994)	47	UK	Not stated	20–60^c^	Nonspecific	Focus groups	Not stated	4
Naish *et al.* (1994)	48	UK	Not stated	Not stated	BAME women	Focus groups	Not stated	6
Ogunsiji *et al.* (2013)	49	Australia	21	25–50	West African immigrant women	Semistructured interviews	Constant comparison	8
Oscarsson *et al.* (2008)	50	Sweden	14	33–64	Women with no cervical smear attendance in previous 5 years	Interviews	Inductive content analysis	8
Park *et al.* (2006)	51	Korea	23	27–37	Sexually active women aged under 40 years	Focus groups	Content analysis	8
Peters (2010)^f^	52	Australia	9	30–65	Women residing in a socioeconomically disadvantaged area	Conversational interviews	Feminist approach	8
Peters (2012)^f^	53	Australia	6	Not stated	Socially disadvantaged women (i.e. from a minority cultural group, had a physical disability and/or had experienced sexual abuse)	Conversational interviews	Feminist approach	7
Power *et al.* (2009)	54	Australia	13	22–42	Lesbian and bisexual women	In‐depth interviews	Thematic analysis	7
Savage and Clarke (1998)	55	Australia	20	46–69	Women aged 45–70 years	Unstructured interviews	Not stated	7
Stewart and Thistlethwaite (2010)	56	Australia	24	18–51	Nonspecific	Semistructured interviews	Deductive content analysis	5
Szarewski *et al.* (2009)	57	UK	28	21–65	Muslim women	Focus groups	Framework analysis	8
Team *et al.* (2013)	58	Australia	8	Min. 40	Russian immigrant women, with caregiving responsibilities	Semistructured interviews	Grounded theory	8
Thomas *et al*. (2005)	59	UK	85	20–75^e^	BAME women	Focus groups + telephone interviews	Content analysis	8
Waller *et al*. (2012)	60	UK	46	25–64	Cervical screening never and irregular attenders	Focus groups + interviews	Framework analysis	8
Widmark *et al*. (2008)	61	Sweden	49	21–74	Nonspecific	Focus groups	Interpretive description	8
Wollin and Elder (2003)	62	Australia	13	Not stated	Deaf women	Interviews	Not stated	7

BAME, Black and Asian minority ethnic; CASP, critical appraisal skills programme.

a Some studies also included data from noneligible participants, that is, health professionals or men. Information on these participants has not been included in this table.

b These three articles are based upon the same sample of women but report different aspects of the data.

c Eligibility age range given as sample age range not reported.

d Includes secondary analysis of Blomberg *et al.* 30.

e Refers to both male and female participants, as separate figures are not reported.

f These two articles are based on the same sample of women but report on different aspects of the data.

### Themes

**Figure 1:**
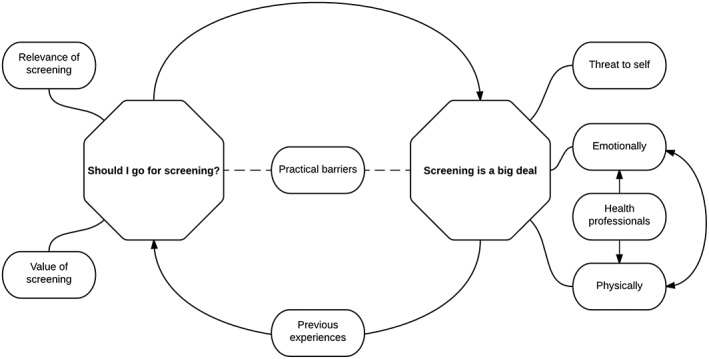
Relationship between identified themes

Two overarching themes were identified: (a) should I go for screening? and (b) screening is a big deal. Figure 1 represents these themes and the main subthemes influencing them. Cervical screening is not a one‐off event but a behaviour that is repeated at regular intervals over decades, so many of the themes discussed were not singular considerations or experiences but were reassessed over time. For further illustrative quotations, see Supporting Information 5.

### Should I go for screening?

The first major theme related to women's thoughts about who needs screening and, consequently, whether they should go for screening. Most women considered whether they should go for screening in relation to the following: (a) the relevance and (b) the value of screening.

#### The relevance of screening – who is it for?

Most studies discussed beliefs regarding who needs to be screened. For some women, the prospect of cervical cancer was considered unlikely, and screening was given little further thought: ‘I just don't believe that it will happen to me and it's not of concern to me’ [P] 49.^2^ Among women who had considered screening, its relevance was largely influenced by four subthemes (causal beliefs, life stage, current health state and family history), and beliefs about relevance appeared to fluctuate over time.

##### Causal beliefs

Discussion about who needs screening was heavily influenced by beliefs about the causes of cervical cancer. Discussions about the numbers of sexual partners and perceptions of promiscuity predominated, but while many women were aware of the link between cervical cancer and sexual behaviour, they were often unclear about the underlying mechanisms. Women often related the risk of cervical cancer to their current sexual behaviour, often emphasising the stability and length of their current relationships, positioning themselves as being at low risk and therefore not needing screening. For BAME women, factors including religion, ethnicity and marital status were seen as synonymous with low‐risk sexual behaviour indicating that screening was unnecessary.

##### Life stage

Many women felt that their age and general feeling of mortality were indicators for screening: ‘Sickness was described as something that could occur at older ages, but not at age 30 years’ [A] 30. For others, reproductive stages and social roles such as motherhood determined screening relevance; for some women, considering having children made them think about their gynaecological health. Menopause was also a time in which the relevance of screening changed. Some women reported feeling more vulnerable during the menopause. For others, being post‐menopausal meant that screening was no longer considered important, having been ‘more essential for them in younger days’ [A] 50.

##### Current health state

Some studies discussed how participants' general feelings of being healthy influenced their perceived need for screening. Other women explicitly discussed the presence or absence of symptoms as an indicator that medical help including screening was needed. Interestingly, this was raised regardless of age, ethnicity or attendance status. For some, lack of symptoms was clearly stated as a reason for nonattendance: ‘If there was anything wrong I think I'd have a discharge or something so I am not so worried about that either’ [P] 61. Other women acknowledged that cancer could be asymptomatic.

##### Family history

Family history was often identified as a risk factor for cervical cancer, and women interpreted its absence as an indication that screening was less important for them.

#### The value of screening – what is the point?

In assessing the value of screening, women fell into one of three groups: those who felt that screening had value, those who did not and those who were aware of screening but unsure of its importance. These assessments were informed by beliefs regarding who needs to be screened, as well as beliefs about the consequences of cervical cancer.

##### Screening has value

Women who believed that screening had value expressed the view that it allowed cancer to be detected early, which was beneficial. These women also valued the reassurance that a negative screening result could provide, giving them ‘peace of mind’ [A/P] 33, 37, 43. There was evidence that some women did not understand the limitations of screening and felt that a negative result provided a ‘certificate of health’ [A] 59, that there was ‘nothing untoward happening’ [P] 26 and that they were ‘free of cancer’ [A] 56. These statements seemed to relate to misperceptions about the purpose of screening, with women seeing it as a general cancer test, test for infections or a reproductive checkup.

Screening was also valued because it allowed women's knowledge of the health of a hidden part of their body: ‘The cervix is an organ inside my body that I cannot see. I can't tell whether it is normal or abnormal, unlike my face, or my hand that I see everyday’ [P] 42.

##### Screening does not have value

A second group of women believed that screening was not important. Some felt that they would know if they were ill or that if there was something wrong, it would resolve by itself. Some described a lack of trust in the test results, potentially based on experience of false positives. For others, there was a general cynicism about the motives of cervical screening programmes, with women suggesting that they were being ‘used to fulfil quotas’ [A] 31 and that screening programmes were ‘trying to get women all in one mind’ [P] 33.

##### Unsure of the importance of screening

The final group appeared to have no opinion of the value of screening. These women had heard of screening but had never considered it to be important. The studies that identified this group were predominantly with women from BAME or lower socioeconomic status backgrounds.

### Screening is a big deal

The second major theme related to women's perceptions that screening is a big deal, both physically and emotionally. This theme relates to perceptions of cervical screening as posing a threat and negative experiences of the procedure.

#### Cervical screening as a threat

##### Potential for screening to reveal cancer

For some women, cervical screening was seen as a significant and emotional experience because of the potential for harm to occur to themselves; the most obvious being a diagnosis of cervical cancer. Fear and anxiety stemming from the potential for cervical screening to identify cancer were related to ideas of pain, suffering and death, as well as concerns about the impact upon fertility among younger women.

##### Screening causes physical harm

Some women worried about clinic hygiene, leading to concern about acquiring an infection or even cervical cancer. With the exception of one study based in Korea 51, this theme was only found in studies with BAME women. For some, screening was considered harmful because it could lead to further investigation or unnecessary treatment.

##### Screening causes anxiety

A more widespread concern was that screening could cause ill health by increasing anxiety and worry, causing an emotional imbalance or making individuals ruminate on the possibility of cancer. For others, the wait for results or threat of a positive result made them anxious.

##### Screening causes a social threat

By attending screening and creating the possibility that they may receive a positive result, women risked facing ‘public distaste and fear of labelling as promiscuous’ [A] 46. This was also related to cultural beliefs about sex before marriage where some women described how they would ‘bin the screening invitation letters to prevent suspicion’ [P] 59. It was acknowledged that some unmarried women were sexually active, often without their parents' knowledge, and that fear of parental disapproval could be a barrier to attendance.

#### The procedure – physical and emotional experiences

The actual procedure was often considered physically and emotionally significant. For some women, cervical screening was simply another health check that needs to be carried out, and while not pleasant, it was not seen as a problem: ‘it's not worse than going to the dentist’ [P] 39. Other women described the test as ‘awful’ [P] 36, ‘pretty revolting’ [P] 55, ‘intrusive’ [A] 24 and ‘daunting’ [A] 47. Women also seemed to be influenced by stories of unpleasant experiences shared by others, especially those who had never attended screening as they did not have access to positive experiences that might counteract negative accounts.

##### Physical experiences

Cervical screening was described as a highly embodied experience. Some women described the procedure as uncomfortable or even painful and reported side effects of the test, including lasting pain and bleeding.

Women also commented on two aspects of the procedure, which they found particularly unpleasant: the taking of the sample and, in particular, the speculum: ‘I don't like the metal thingamajig they use actually. I think that's what puts me off more than anything’ [P] 60. Discussion of the speculum also related its coldness and the act of penetration.

Some women had a good understanding of how the sample was taken but felt highly aware of what had been done to their bodies. Other women had misconceptions about the procedure, believing that the cervix was ‘frozen’ [P] 30 or that ‘pieces are cut from the womb with the speculum’ [A] 48.

##### Emotional experiences

Women reported strong emotional responses to the procedure, including embarrassment, shame and vulnerability. Some perceived the test as degrading and violating and described a loss of power and control over the situation. Experiencing negative emotions during the procedure also worsened the physical experience for some women, through increasing tension.

Negative emotional experiences were also associated with events outside the actual appointment. Some women described how receiving an invitation resulted in anxiety, and they felt that it was necessary to ‘psych themselves up’ [A] 47 or ‘pluck up the courage’ [P] 26 for the encounter.

Many of the negative emotions women described involved embarrassment and shame, stemming from a situation in which social norms surrounding nudity are broken: ‘Even though she is a female doctor but I was still ashamed the first time I did it, of opening up my private part to somebody, but after that I got used to it’ [P] 49. Some older women and women who had experienced abuse had particular concerns about the appearance of their body, which made them reluctant to attend. Some Somali women, although not ashamed of it, were worried about the smear taker's response to the fact that they had been circumcised and considered that it may cause further embarrassment. There was particular concern from some BAME women about exposing their bodies, because of the belief that their naked body should only be seen by their husband.

Concerns about nudity and the sexual connotations of the procedure resulted in a strong preference across studies for female smear takers. Although this was often still seen as embarrassing, seeing a male health professional resulted in the most severe negative emotional reactions: ‘I hate the idea of going to a man doctor. It's a lot of embarrassment for me. I'm not going back to him. Even though it lasts only a couple of minutes I think it's, it's very undignified’ [P] 44.

Some women also believed that a female smear taker would be more sympathetic to women's health issues and that having experienced it themselves, they would be more likely to be gentle and considerate.

##### Health professionals

Women's experiences of screening were often shaped by the quality of their interaction with the health professional carrying out the procedure. Some women reported positive encounters with these health professionals, but more commonly, women recalled negative encounters, with poor communication often cited as the reason.

Women expressed a desire for explanation about the procedure as it is carried out and the opportunity to ask questions, but many reported negative experiences where this ideal had not been met. Poor communication seemed to exacerbate the loss of control that some women associated with screening. Where clear information about the procedure was provided, women reported much more satisfactory experiences.

Some women reported situations in which they felt that their experience of the test had been dismissed or ignored by the smear taker. Others described inconsiderate or disrespectful behaviour by the smear taker, including ‘ignorance, rough treatment, insensitivity… joking, crudeness and shouting at [the] patient’ [P] 35. Further, BAME women and women with disabilities sometimes found smear takers to be prejudiced or insensitive to their specific needs, and both groups suggested specialised training to improve clinical encounters.

Efforts by health professionals to reduce or deny the emotional significance of the procedure by emphasising its routine nature were largely disliked. In doing so, women felt that the smear taker was disregarding the significance it held for them: ‘Doctors may perform the same test many times, but it is not such a routine test to an individual woman’ [P] 51. In particular, women wanted the smear taker to acknowledge the emotional significance that cervical screening held for them. In some cases, women found the experience de‐individualising, especially when the smear taker emphasised that they'd ‘seen it all before’: ‘Doctors have seen a lot of vaginas but they didn't see mine and that's it for me. […] So I just saw it as an invasion of my person and my privacy’ [P] 27. In some cases, women used language that suggests that they found the experience to be dehumanising, referring to feeling like ‘a piece of meat’ [P] 31, 40 and being ‘treated like…cattle’ [P] 28.

##### Smear taker preferences

In addition to a female smear taker (see previous text), women expressed other preferences, which they hoped would improve the screening experience. Some were concerned about having a trusting relationship with the smear taker, which usually meant seeking continuity of care. Others preferred the relative anonymity of having cervical screening carried out by an unfamiliar health professional. Less commonly, women expressed preferences regarding professional background.

The BAME women sometimes expressed a preference for health professionals who were of the same ethnic background. Others preferred to visit health professionals of a different background, as a strategy to avoid potential breaches of confidentiality.

### Additional contributing factors

In addition to the two main themes identified, some minor themes played additional roles. These included the following: (a) previous experiences and (b) practical barriers.

#### Previous experiences

Previous experiences included general experiences in healthcare settings as well as more specific experiences with screening.

##### Experiences with healthcare provision in general

Experiences of healthcare outside of screening, in particular gynaecological examinations, influenced intentions to have cervical screening. A single negative experience of a gynaecological examination could result in women avoiding screening. Other experiences of healthcare, such as breaches of confidentiality, affected trust in the system and willingness to attend screening.

##### Previous experiences of cervical screening

Previous screening experiences seemed to influence decisions regarding re‐attendance in two ways. Firstly, previous normal results were sometimes interpreted as being given an ‘all clear’ with no further need to attend. Conversely, one woman reported how a previous positive result reduced her desire to attend screening because of the increased anxiety that she now felt.

Secondly, even when screening was still considered relevant, previous experiences influenced willingness to re‐attend. For some women, a single negative experience prevented them from re‐attending screening, even if they had multiple positive previous experiences to draw upon.

#### Practical barriers

Women reported a number of practical barriers to accessing screening, which were often associated with their life circumstances and the resources available to them. Some of these women wanted to attend screening but felt prevented by practical barriers. For others, practical barriers were indicated as reasons for nonattendance in addition to one or more of the barriers outlined previously.

Women described how screening was yet another demand on their time and often competed with daily tasks such as work and childcare, which were given higher priority. For others, screening was seen as inaccessible, because of features of the clinic such as inconvenient location or appointment times.

There were also specific barriers reported by BAME women. Some women felt that they had received racist treatment by health professionals, which made them less willing to attend. Others reported difficulties in accessing information. For some women, this was due to low competency in the language of their country of residence and a lack of translated materials. For others, low literacy levels or a cultural preference for verbal information meant that they could not access information even when translated. The impact of these factors was largely related to the screening context of women's country of origin. Women from Somalia 24 and west Africa 49 reported being unaware of cervical screening before migrating, while women from eastern Europe 41, 58 had prior knowledge of screening because of participating in their home country.

## Conclusions

Through synthesising the qualitative research on women's perceptions and experiences of cervical screening, we identified two novel overarching themes; the first, ‘should I go for screening?’, is indicative of women who are undecided about attending screening, after the first invitation and on subsequent occasions, as they consider both the relevance and the value of screening. The second theme, ‘screening is a big deal’, is reflective of the experiences of women who have already attended screening at least once, relating in particular to the ways in which screening presented a threat, and the largely negative physical and emotional experiences of the procedure. These negative experiences were both inherent to the procedure and influenced by the conduct of health professionals. As screening is not a one‐time event but an action that should be repeated at regular intervals over decades, negative experiences clearly inform women's decisions about future attendance.

There were also instances of women who wished to attend but had difficulty translating those intentions into action because of competing priorities and practical barriers and the resources available to overcome these. As well as women who intended to act and those who were undecided, there were also women who had made an active decision to not attend screening, although this does not necessarily mean that they had not previously attended or would definitely not attend in the future. It is unclear whether these women are making an informed choice not to attend or are basing their decisions on lack of understanding or misconceptions.

We also identified women within the synthesis who were unaware of or unengaged with screening, although these themes were far less developed than those previously outlined. It is clear that these perspectives are much less explored within the literature than others, as the majority of women across studies had attended screening at least once. One reason for this may be the way in which women are recruited for studies. It is unlikely that unaware or unengaged women would be motivated to take part in a study about screening. As this may be a target group for interventions aimed at increasing engagement with the screening invitation, further research may need to seek a different method of recruitment and approach to the subject of screening in order to ensure that the perspectives of these groups are included in intervention design and evaluation.

These findings show that there are a number of reasons why women may not attend cervical screening as recommended and highlight the fact that screening nonattenders should not be viewed as a homogenous group. There may be value in considering different subgroups and the associated reasons for nonattendance, particularly when developing interventions to promote uptake. Stage‐based models of behaviour change, such as the transtheoretical model (TTM) 63 and the precaution adoption process model (PAPM) 64, offer ways of conceptualising these differences in engagement with cervical screening. Findings from this synthesis suggest that the PAPM, with its more detailed categorisation of those with no intention to act into ‘unaware’, ‘unengaged’ and ‘decided not to act’ groups, may be of greater value than the TTM with its singular ‘precontemplation’ stage, when considering ways to support women in making an informed choice about cervical screening. Previous studies of mammography 65, 66 and colorectal cancer screening 67, 68, 69 readiness have shown that the stages of the PAPM are associated with particular sociodemographic and psychological correlates, which could be used to inform the development of targeted interventions. While our findings offer support for the value of using the PAPM to categorise women in terms of their engagement with cervical screening, further research is needed to establish the proportion of women within each group in the general population and to examine stage‐specific differences in sociodemographic and psychological characteristics.

Our identification of the themes of ‘personal relevance’ and ‘value’ in women's decision‐making is a new way of conceptualising this stage of the process and can now be used to develop appropriate information for women deciding whether to take part. Designing information to address the issues that women across studies grapple with may be a way to make it more relevant and helpful. In addition, our finding that ‘screening is a big deal’ was such a major theme in women's accounts speaks to a strong need to acknowledge this within programmes and to develop ways to minimise negative physical and psychological consequences.

### Limitations

Inherent to qualitative synthesis are the limitations that arise from working with published articles. It is impossible to know whether the reported data and authors' interpretations were representative of the data as a whole or the extent to which reported quotes were spontaneous from participants. Further, through the process of synthesis, the specific context of studies – a valuable component of qualitative research – may be compromised. Although we attempted to remain mindful of this issue and ensure that themes were not inappropriately presented as universal, it is inevitable that some context and richness of data will have been lost in the process of translating and synthesising across studies.

There are also limitations specific to this synthesis. Although our search strategy included a number of other countries, UK‐based and Australia‐based (and, therefore, Anglophone) studies dominated the final sample. That the same themes were also found in the smaller number of studies from Sweden and Korea supports the idea that our findings might apply to other countries with similar screening programmes, but it is possible that women's perceptions of cervical screening may differ in other contexts. There is also a need for further research to ensure that the perspectives of women who have never attended are included.

### Implications

This review points to some clear ways for practitioners to minimise the negative aspects of the screening experience, for example, providing the option of a female smear taker, acknowledging that the procedure is not experienced as routine for women, taking steps to ensure that dignity is maintained and being sensitive to the specific needs of women from BAME groups, women with disabilities and those who have experienced abuse. The wide variety of barriers to screening identified makes it clear that nonparticipants are a heterogeneous group. Conceptualising screening nonparticipants along a continuum of screening adoption may be a useful way of targeting appropriate interventions, from awareness‐raising initiatives for women who are unaware of screening in order to promote informed choice about screening to addressing practical barriers for women who have difficulty translating positive intentions into attendance. Our findings identify the need for more research to understand how and why some women in countries with established screening programmes remain unaware of screening. There is some evidence that BAME women may be more likely to be unaware or unengaged 70, but the issue should be explored further with a view to reducing social inequalities in screening participation.

### Summary

To our knowledge, this is the first time that the methods of thematic synthesis have been applied to studies on women's perceptions of cervical screening. Doing so identified two broad themes in women's narratives of cervical screening, which were not wholly apparent in any given study. Many women considered both the personal relevance and the value of screening when deciding whether they should attend. Those who had previously been screened reported largely negative experiences of screening, which informed their decisions about future attendance. We also identified practical barriers to attendance, which may prevent women from realising their screening intentions. In addition to these themes, drawing together multiple studies highlighted the scarcity of accounts from women who have never attended screening and identified the need for further research. Combined, these findings highlight the varying reasons that women may have for nonattendance and suggest that interventions tailored to decisional stage may be of value in increasing informed uptake of cervical screening.

## Conflict of interest

The authors have no conflicts of interest to declare.

## Supporting information

Supporting info item

Supporting info item

Supporting info item

Supporting info item

Supporting info item

## Notes

1 Age‐appropriate coverage refers to the percentage of screening‐eligible women in the population who were screened within 3.5 years for those aged 25–49 years and within 5.5 years for those aged 50–64 years.

2 [P] denotes a participant comment; [A] denotes an author comment.
