# Supplementary material for: Experiences of cervical screening and barriers to participation in the context of an organised programme: a systematic review and thematic synthesis
Source: Psychooncology. 2016 Apr 12;26(2):161–72. doi: 10.1002/pon.4126 (PMC5324630; doi:10.1002/pon.4126)
Supplement: Supplementary file 1 — Supporting info item [file PON-26-161-s001.doc]

#### Table 1. Countries with established cervical screening programmes, included in searches

|  | **Year of programme initiation** | **Age range** | **Recommended frequency** | **Number of studies included in review** |
| --- | --- | --- | --- | --- |
|  |  |  |  |  |
| Denmark | 1962 | 23 – 65 | 3 years (23 – 50)  5 years (51 – 65) | 0 |
| Finland | 1963 | 30 – 65 | 5 years | 0 |
| Iceland | 1964 | 20 – 69 | 2 years (20 – 39)  4 years (40 – 69) | 0 |
| Sweden | 1967 | 23 – 60 | 3 years (23 – 50)  5 years (51 – 60) | 7 |
| Korea | 1988 | 30 – 70+ | 2 years | 1 |
| United Kingdom | 1988 | 25 – 64 | 3 years (25 – 49)  5 years (50 – 64) | 20 |
| Netherlands | 1989 | 30 – 60 | 5 years | 0 |
| Australia | 1991 | 18 – 69  (or 2 years after first intercourse) | 2 years | 11 |
| Norway | 1995 | 25 - 69 | 3 years | 0 |
| Slovenia | 2003 | 20 – 64 | 3 years | 0 |

Note: Canada, Italy and New Zealand were also included in the initial search criteria, but studies from these countries were later excluded due to the organised screening programmes not being nationwide (Canada and Italy) or not having an initial invitation system for all eligible women (New Zealand).
