# Supplementary material for: Experiences of cervical screening and barriers to participation in the context of an organised programme: a systematic review and thematic synthesis
Source: Psychooncology. 2016 Apr 12;26(2):161–72. doi: 10.1002/pon.4126 (PMC5324630; doi:10.1002/pon.4126)
Supplement: Supplementary file 2 — Supporting info item [file PON-26-161-s002.doc]

|  | **MEDLINE** | **PsycINFO** | **Embase** | **Social Policy and Practice** | **CINAHL Plus** | **AnthroSource** | **ProQuest Social Science Journals** | **POPLINE** | **Web of Science** |
| --- | --- | --- | --- | --- | --- | --- | --- | --- | --- |
| **Cervical Screening** | exp Vaginal Smears/  exp Papanicolaou Test/  “vaginal smear”.ab,ti.  “papanicolaou”.ab,ti.  “cervical screening”.ab,ti.  “liquid based cytology”.ab,ti.  “cervical cancer screening”.ab,ti.  “smear test”.ab,ti.  “pap”.ab,ti.  “hpv test”.ab,ti. | exp Vaginal Smears/  exp Papanicolaou Test/  “vaginal smear”.ab,ti.  “papanicolaou”.ab,ti.  “cervical screening”.ab,ti.  “liquid based cytology”.ab,ti.  “cervical cancer screening”.ab,ti.  “smear test”.ab,ti.  “pap”.ab,ti.  “hpv test”.ab,ti. | exp Vaginal Smears/  exp Papanicolaou Test/  “vaginal smear”.ab,ti.  “papanicolaou”.ab,ti.  “cervical screening”.ab,ti.  “liquid based cytology”.ab,ti.  “cervical cancer screening”.ab,ti.  “smear test”.ab,ti.  “pap”.ab,ti.  “hpv test”.ab,ti. | exp Vaginal Smears/  exp Papanicolaou Test/  “vaginal smear”.ab,ti.  “papanicolaou”.ab,ti.  “cervical screening”.ab,ti.  “liquid based cytology”.ab,ti.  “cervical cancer screening”.ab,ti.  “smear test”.ab,ti.  “pap”.ab,ti.  “hpv test”.ab,ti. | (MH "Cancer Screening")  (MH "Papillomaviruses")  (MH "Cervical Smears")  "cervical screening" "cervical cancer screening"  "cervical smear*"  "liquid based cytology"  "smear test"  "pap test"  "papanicolaou”  "hpv test*" | “cervical”  “pap”  “smear”  “HPV”  “cytology” | "cervical screening" "vaginal smear" "cervical cancer screening"  "smear*"  "papanicolaou"  "pap test" "liquid based cytology"  "hpv test" | "cervical screening" "smear*"  "pap test"  “papanicolaou”  "hpv test"  "cervical cancer" "liquid based cytology" "vaginal smear" | "vaginal smear*"  “papanicolaou”  "pap test"  "smear"  "cervical screening"  "cervical cancer screening"  "liquid based cytology"  "hpv test" |
|  | AND | AND | AND | AND |  | AND |  |  | AND |
| **Qualitative** | exp Qualitative Research/  exp Focus Groups/  exp Interview/  “qualitative”.ab,ti.  “focus groups”.ab,ti.  “interview*”.ab,ti.  “grounded theory”.ab,ti.  “ethnogr*”.ab,ti.  “participant observation”.ab,ti.  “thematic analysis”.ab,ti.  “phenomenolog*”.ab,ti.  “content analysis”.ab,ti.  “framework analysis”.ab,ti. | exp Qualitative Research/  exp Focus Groups/  exp Interview/ “qualitative”.ab,ti.  “focus groups”.ab,ti.  “interview*”.ab,ti.  “grounded theory”.ab,ti.  “ethnogr*”.ab,ti.  “participant observation”.ab,ti.  “thematic analysis”.ab,ti.  “phenomenolog*”.ab,ti.  “content analysis”.ab,ti.  “framework analysis”.ab,ti. | exp Qualitative Research/  exp Focus Groups/  exp Interview/ “qualitative”.ab,ti.  “focus groups”.ab,ti.  “interview*”.ab,ti.  “grounded theory”.ab,ti.  “ethnogr*”.ab,ti.  “participant observation”.ab,ti.  “thematic analysis”.ab,ti.  “phenomenolog*”.ab,ti.  “content analysis”.ab,ti.  “framework analysis”.ab,ti. | exp Qualitative Research/  exp Focus Groups/  exp Interview/  “qualitative”.ab,ti.  “focus groups”.ab,ti.  “interview*”.ab,ti.  “grounded theory”.ab,ti.  “ethnogr*”.ab,ti.  “participant observation”.ab,ti.  “thematic analysis”.ab,ti.  “phenomenolog*”.ab,ti.  “content analysis”.ab,ti.  “framework analysis”.ab,ti. | (MH "Qualitative Studies+")  (MH "Focus Groups") (MH "Interviews+") (MH "Semi-Structured Interview")  (MH "Structured Interview")  (MH "Unstructured Interview")  (MH "Narratives")  (MH "Observational Methods+")  (MH "Nonparticipant Observation")  (MH "Participant Observation")  (MH "Grounded Theory")  (MH "Content Analysis")  (MH "Thematic Analysis")  (MH "Phenomenological Research")  (MH "Conceptual Framework")  "qualitative"  "focus group*"  "interview*"  "grounded theory"  "ethnogr*"  "participant observation" "thematic analysis" "phenomenolog*"  "content analysis"  "framework analysis" "anthrop*" |  | "qualitative"  "focus group*"  "interview*"  "grounded theory"  "ethnogr*"  "participant observation" "thematic analysis" "phenomenolog*"  "content analysis"  "framework analysis" "anthrop*" | "qualitative" "focus group*" "interview*" "grounded theory" "ethnogr*" "participant observation" "thematic analysis" "phenomenology*" "content analysis" "framework analysis" "anthrop*" | "qualitative"  "focus group*"  "interview*"  "grounded theory"  "ethnogr*"  "participant observation" "thematic analysis" "phenomenolog*"  "content analysis"  "framework analysis" "anthrop*" |
|  | AND | AND | AND | AND | AND | AND |  |  | AND |
| **Barriers** | exp. Attitude/  exp Health Knowledge, Attitudes, Practice/  exp. Health Behaviour/  exp. Social Perception/  exp. Patient Acceptance of Healthcare/  exp. Attitude to Health/  “attitude*”.ab,ti.  “belief*”.ab,ti.  “reasons”.ab,ti.  “perception*”.ab,ti.  “barriers”.ab,ti. | exp. Attitude/  exp Health Knowledge, Attitudes, Practice/  exp. Health Behaviour/  exp. Social Perception/  exp. Patient Acceptance of Healthcare/  exp. Attitude to Health/  “attitude*”.ab,ti.  “belief*”.ab,ti.  “reasons”.ab,ti.  “perception*”.ab,ti.  “barriers”.ab,ti. | exp. Attitude/  exp Health Knowledge, Attitudes, Practice/  exp. Health Behaviour/  exp. Social Perception/  exp. Patient Acceptance of Healthcare/  exp. Attitude to Health/  “attitude*”.ab,ti.  “belief*”.ab,ti.  “reasons”.ab,ti.  “perception*”.ab,ti.  “barriers”.ab,ti. | exp. Attitude/  exp Health Knowledge, Attitudes, Practice/  exp. Health Behaviour/  exp. Social Perception/  exp. Patient Acceptance of Healthcare/  exp. Attitude to Health/  “attitude*”.ab,ti.  “belief*”.ab,ti.  “reasons”.ab,ti.  “perception*”.ab,ti.  “barriers”.ab,ti. | (MH "Attitude")  (MH "Health Behavior")  (MH "Health Knowledge")  ""attitud*""  (MH "Health Beliefs")  (MH "Attitude to Health")  ""belief*""  ""reason*""  ""perception*"" ""barrier*"" |  | "attitud*"  "belief*"  "behaviou*"  "reason*"  "perception"  "barrier*" | "attitud*" "belief*" "behavio*" "reason*" "perception" "barrier*" | "attitud*"  "belief*"  "behaviou*"  "reason*"  "perception"  "barrier*" |
|  | AND | AND | AND | AND |  | AND |  | AND | AND |
| **Countries** | exp Australia/  exp Canada/  exp Denmark/  exp Finland/  exp Iceland/  exp Italy/  exp “Republic of Korea”/  exp Netherlands/  exp New Zealand/  exp Norway/  exp Slovenia/  exp Sweden/  exp Great Britain/ or Channel Islands/ or England/ or Northern Ireland/ or Scotland/ or Wales/  “Australia”.ab,ti.  “Canada”.ab,ti.  “Denmark”.ab,ti.  “Finland”.ab,ti.  “Iceland”.ab,ti.  “Italy”.ab,ti.  “Korea”.ab,ti.  “Netherlands”.ab,ti.  “New Zealand”.ab,ti.  “Norway”.ab,ti.  “Slovenia”.ab,ti.  “Sweden”.ab,ti.  “Great Britain”.ab,ti.  “Channel Islands”.ab,ti.  “United Kingdom”.ab,ti.  “England”.ab,ti.  “Northern Ireland”.ab,ti.  “Scotland”.ab,ti.  “Wales”.ab,ti. | exp Australia/  exp Canada/  exp Denmark/  exp Finland/  exp Iceland/  exp Italy/  exp “Republic of Korea”/  exp Netherlands/  exp New Zealand/  exp Norway/  exp Slovenia/  exp Sweden/  exp Great Britain/ or Channel Islands/ or England/ or Northern Ireland/ or Scotland/ or Wales/  “Australia”.ab,ti.  “Canada”.ab,ti.  “Denmark”.ab,ti.  “Finland”.ab,ti.  “Iceland”.ab,ti.  “Italy”.ab,ti.  “Korea”.ab,ti.  “Netherlands”.ab,ti.  “New Zealand”.ab,ti.  “Norway”.ab,ti.  “Slovenia”.ab,ti.  “Sweden”.ab,ti.  “Great Britain”.ab,ti.  “Channel Islands”.ab,ti.  “United Kingdom”.ab,ti.  “England”.ab,ti.  “Northern Ireland”.ab,ti.  “Scotland”.ab,ti.  “Wales”.ab,ti. | exp Australia/  exp Canada/  exp Denmark/  exp Finland/  exp Iceland/  exp Italy/  exp “Republic of Korea”/  exp Netherlands/  exp New Zealand/  exp Norway/  exp Slovenia/  exp Sweden/  exp Great Britain/ or Channel Islands/ or England/ or Northern Ireland/ or Scotland/ or Wales/  “Australia”.ab,ti.  “Canada”.ab,ti.  “Denmark”.ab,ti.  “Finland”.ab,ti.  “Iceland”.ab,ti.  “Italy”.ab,ti.  “Korea”.ab,ti.  “Netherlands”.ab,ti.  “New Zealand”.ab,ti.  “Norway”.ab,ti.  “Slovenia”.ab,ti.  “Sweden”.ab,ti.  “Great Britain”.ab,ti.  “Channel Islands”.ab,ti.  “United Kingdom”.ab,ti.  “England”.ab,ti.  “Northern Ireland”.ab,ti.  “Scotland”.ab,ti.  “Wales”.ab,ti. | exp Australia/  exp Canada/  exp Denmark/  exp Finland/  exp Iceland/  exp Italy/  exp “Republic of Korea”/  exp Netherlands/  exp New Zealand/  exp Norway/  exp Slovenia/  exp Sweden/  exp Great Britain/ or Channel Islands/ or England/ or Northern Ireland/ or Scotland/ or Wales/  “Australia”.ab,ti.  “Canada”.ab,ti.  “Denmark”.ab,ti.  “Finland”.ab,ti.  “Iceland”.ab,ti.  “Italy”.ab,ti.  “Korea”.ab,ti.  “Netherlands”.ab,ti.  “New Zealand”.ab,ti.  “Norway”.ab,ti.  “Slovenia”.ab,ti.  “Sweden”.ab,ti.  “Great Britain”.ab,ti.  “Channel Islands”.ab,ti.  “United Kingdom”.ab,ti.  “England”.ab,ti.  “Northern Ireland”.ab,ti.  “Scotland”.ab,ti.  “Wales”.ab,ti. | (MH "Australia") "australia"  (MH "Canada")  "canada"  (MH "Denmark")  "denmark"  "finland"  (MH "Iceland") "iceland"  (MH "Italy")  "italy"  (MH "Korea")  "korea"  (MH "South Korea") (MH "Netherlands") "netherlands"  (MH "New Zealand") ""new zealand""  (MH "Norway") "norway"  (MH "Slovenia")  "slovenia"  (MH "Sweden")  "sweden"  (MH "Great Britain") (MH "England")  (MH "Scotland")  (MH "Wales")  (MH "Northern Ireland")  (MH "United Kingdom")  ""great britain""  ""channel islands"" ""united kingdom"" ""england"" ""northern ireland"" ""scotland"" ""wales"" |  | "australia"  "canada"  "denmark"  "finland"  "iceland"  "italy"  "korea"  "netherlands"  "new zealand"  "norway"  "slovenia"  "sweden"  "great britain"  "channel islands"  "united kingdom"  "england"  "northern ireland" "scotland"  "wales" | "australia" "canada" "denmark" "finland" "iceland"  "italy"  "korea"  "netherlands" "new zealand" "norway" "slovenia" "sweden"  "great britain" "channel islands"  "united kingdom" "england" "northern ireland" "scotland" "wales" | "australia"  "canada"  "denmark"  "finland"  "iceland"  "italy"  "korea"  "netherlands"  "new zealand"  "norway"  "slovenia"  "sweden"  "great britain"  "channel islands"  "united kingdom"  "england"  "northern ireland" "scotland"  "wales" |
| **Number of hits[[1]](#footnote-2)** | 167 | 26 | 197 | 1 | 214 | 14 | 5 | 14 | 206 |

1. As of 12^th^ February 2015 [↑](#footnote-ref-2)
