# Supplementary material for: Experiences of cervical screening and barriers to participation in the context of an organised programme: a systematic review and thematic synthesis
Source: Psychooncology. 2016 Apr 12;26(2):161–72. doi: 10.1002/pon.4126 (PMC5324630; doi:10.1002/pon.4126)
Supplement: Supplementary file 3 — Supporting info item [file PON-26-161-s003.doc]

#### Box 1. Exclusion criteria for coded text

| 1. Text directly relates to an outcome other than cervical screening, e.g. mammography screening, women’s health in general, etc. 2. An introduction paragraph that summarises data described in detail later in the results. 3. Statements describing the screening context. 4. Descriptive sentences about the number of women participating in screening. 5. Interpretive sections of text including references to other studies rather than original data. 6. References to quantitative data where mixed methods were used. 7. References to data from health professionals where mixed-samples were used. 8. References to themes described by women with abnormal results where this relates directly to the experience of receiving an abnormal result. 9. Text that is unclear/vague and could therefore not be coded meaningfully. |
| --- |
