# Supplementary material for: Experiences of cervical screening and barriers to participation in the context of an organised programme: a systematic review and thematic synthesis
Source: Psychooncology. 2016 Apr 12;26(2):161–72. doi: 10.1002/pon.4126 (PMC5324630; doi:10.1002/pon.4126)
Supplement: Supplementary file 4 — Supporting info item [file PON-26-161-s004.doc]

**Flow diagram of study selection**

^1^Our initial search strategy included Canada, Italy, and New Zealand, but these countries were later excluded due to not having an eligible population-wide call-recall screening programme. Due to this, the number of exclusion at this stage is higher than expected.

Records excluded

(n = 71)

Not about screening = 17

Ineligible participants = 2

Ineligible countries = 36

No qualitative data = 12

Not primary research = 3

No English full-text = 1

Identification

Records identified through database searching

(N = 844)

MEDLINE n = 167

PsycINFO n = 26

Embase n = 197

Social Policy and Practice n = 1

CINAHL Plus n = 214

ProQuest Social Science Journals n = 5

Anthrosource n = 14

POPLINE n = 14

Web of Science n = 206

Screening

Titles and abstracts screened

(n = 450)

Records identified through handsearching

(n = 7)

Records excluded

(n = 347)1

Eligibility

Full text articles assessed for eligibility

(n = 103)

Studies included in qualitative synthesis

(n =39)

Included

Duplicates removed

(n = 394)
