# Supplementary material for: Experiences of cervical screening and barriers to participation in the context of an organised programme: a systematic review and thematic synthesis
Source: Psychooncology. 2016 Apr 12;26(2):161–72. doi: 10.1002/pon.4126 (PMC5324630; doi:10.1002/pon.4126)
Supplement: Supplementary file 5 — Supporting info item [file PON-26-161-s005.doc]

| **Main theme** | **Sub-themes** | | **Illustrative quotations** |
| --- | --- | --- | --- |
| Should I go for screening? | The relevance of screening – who’s it for? | Causal beliefs | *“I think only women who are promiscuous would get cervical cancer or cancer of their reproductive organs.”* [P]^1^ [43]  *“Yes, there are those kinds of changes you can get when you change partners, there’s always that, but it hopefully won’t happen [laughs]. We’ve been together for ten years so we can hope it will last [laughs]”* [P] [28] |
|  |  | Life stage | “*Well, if it helps the young ones, because they’ve got a young family. I mean my children have grown up”* [P] [55]  “*I think I’ve also started to think more now than when I was 25, because you think you want to have children sometime, and now you maybe should start to think about it and go to these…[screening programme] and check that everything you’ve taken for granted all these years is OK*” [P] [30]  “*And I think maybe [the menopause] is a very uncertain time for women of our age and maybe it is the most important period of your life to go [attend for screening] through”* [P] [26] |
|  |  | Current health state | “*I think it’s [screening] unnecessary since I don’t smoke and I have a sound and healthy lifestyle”* [P] [28] |
|  |  | Family history | “*Well, it’s quite stupid really because it is important to check. But then I think that there’s no one in my family that has ever had any problems there. […] So there’s no worries*” [P] [50] |
|  | The value of screening – what’s the point? | Screening has value | *“If they catch it in the beginning there is more chance of them treating it”* [P] [57] |
|  |  | Screening does not have value | “*I think I would know if there was something haywire*” [P] [55]  “*I don’t want to go for a test… I don’t trust them. Think if they diagnose me with a cancer and it turns out that I haven’t–I don’t want them to disrupt my mind!”* [P] [38] |
|  |  | Unsure of the importance of screening | *“I didn’t know anything about cervical cancer and that I had to go for cervical screening. . . . I didn’t go because I didn’t know that it was important.”* [P] [44] |
|  | | | |
| Screening is a big deal | Cervical screening as a threat | The potential for screening to reveal cancer | “*I don’t want to know about it really because it’s a frightening thing”* [P] [55] |
|  |  | Screening causes physical harm | “*But the cancer might be there [in the clinic]… you never know… they need to cover it with water, wash it all away… I’ve never seen them do that”* [P] [31] |
|  |  | Screening causes anxiety | “*Some women perceived that thinking about cancer would “cause” cancer to develop, and accordingly, adherence to screening might precipitate rather than prevent the disease*” [A] [42]  “*The worry, which can also elicit sickness, and which the body is exposed to during the wait for the test results”* [P] [28] |
|  |  | Screening causes a social threat | *“They said that a visit to an OB– Gyn clinic had a negative connotation, suggesting that the woman might have a “filthy condition,” possibly related to sexual matters.”* [A] [51] |
|  | The procedure | Physical experiences | *“It felt painful and awkward. It was painful after it was done and I bled afterwards”* [P] [32]  “*I just heard it was uncomfortable and nobody really enjoyed going, so I didn’t really want to go”* [P] [44]  “*But it’s cold and you know that there’s an implement that is pushed in and so that’s painful as well*” [P] [27]  “*I felt sore, I could always feel where they had scraped those cells”* [P] [55] |
|  |  | Emotional experiences | “*I felt so embarrassed, exposed to all, powerless, cold... and it was my body”* [P] [31]  *“When this letter arrived and said I had to go...I was petrified!”* [P] [33]  “*As you get older you get, like your breasts aren’t as ﬁrm as they used to be, your belly starts hanging down and, you know, little bits of you start going out of shape and you think I don’t want anybody looking at me like that”* [P] [26]  “*Also, since we have been circumcised, you’ll be embarrassed to surprise them: What happened to her?”* [P] [24] |
|  |  | Health professionals | “*Only a woman knows how it feels to have a Pap smear…I just like the idea of a woman who’s had it done, to be doing it to me and knowing what it feels like.*” [P] [52]  “*The nurse […] didn’t make me, make me relaxed, you know. I just felt like she just had to do what she had to do and I didn’t know what was happening. Maybe she didn’t prepare me enough.*” [P] [27]  “*The lack of explanation in preparation or during the test had helped engender a feeling of helplessness amongst them.*” [A] [36]  “*They were hurting me and just kind of ignoring me. Not putting any attention on what I was feeling, you know and that it was actually hurting me”* [P] [27]  “*the procedure should not be treated as routine but as an emotional experience for each individual*” [A] [47] |
|  |  | Smear taker preferences | “*It is so embarrassing to have the (Pap) test with my GP. I see him for all sorts of sicknesses but I never thought of having the test with him. He referred me to a female GP for a Pap smear. Whenever I have the Pap, I will go to her. I cannot imagine myself seeing the same male GP after opening my legs to have the Pap test taken by him*” [P] [42]  “Th*e last two times have been a lot better because it’s been performed by the nurse so it’s been a totally different, a totally different experience … not as horrific as it was when the doctor does it”* [P] [27]  “*I don’t have difficulties communicating with doctors in English, but I still prefer having a Chinese speaking doctor for a Pap smear test because I feel best able to express myself and my feelings, particularly during a procedure where I feel very vulnerable already”* [P] [43] |
|  | | | |
| Previous experiences | Experiences with general healthcare provision | | “*It hurt so much that they held me down, that he didn’t stop it then. […] They used force on me, that’s how I felt. I can picture myself as a victim who had to suﬀer torture”*. *This experience greatly inﬂuenced her decision not to attend [screening] and she feared that this could happen again*” [P/A] [50]  “*For example, issues of lack of confidentiality could lead to general distrust of the health-care system and its professionals and an unwillingness to attend [screening]*” [A] [28] |
|  | Previous experiences of cervical screening | | “*I’ve been twice and there is nothing there and now I have no husband because he has died so I have no sexual relation with anyone so after going twice I don’t need them now”* [P] [25]  *“I think it was bad experience in the past, which then led to apathy and … looking for a way out of going through it again, which was the fact that I didn’t think I was high risk”* [P] [60] |
|  | | | |
| Practical barriers | Competing priorities | Work | *Whenever they send me an appointment it never suits me and I can’t get time off work for that. Can you see me asking my boss for time off to go for that?*” [P] [44] |
|  |  | Childcare responsibilities | “*There should be a creche where the children can sleep while the mum is in there*” [P] [24] |
|  |  | Other commitments | “*I’ve got to have blood tests, I’ve got to go to the dentist, I’ve got to get my hair cut, so what’s low in priority, and a cervical smear test would be right down there I think*” [P] [60] |
|  |  |  |  |
|  | Accessibility | Indirect financial costs | “*Taking time off work for screening was difficult, or would mean forgoing income which they could not afford to lose*” [A] [41] |
|  |  | Clinic location | “*I can’t drive and there is no public transport around here. I don’t want to bother my daughter to drive me to the clinic, as the Pap smear test is not that important anyway*” [P] [43] |
|  |  | Inflexible appointment times | “*I think if they were to have clinics on Saturdays or in the evening that they would get more women in*” [P] [44] |
|  | Communication issues | Language barriers | “*I’m not from an English speaking background and we do not know where to go and who to go to and [we are] not always being encouraged to go for it [women’s health screening]*” [P] [53] |
|  |  | Low literacy levels | “*Translated information about the smear test was available but this could not reach the women who were non-literate in any language.*” [A] [31] |
|  |  | Preference for verbal information | “*Somali people don’t respond well to reading… you’ll get sent a letter and just put it down somewhere, not paying too much attention to it. Our knowledge is passed on verbally*” [P] [24] |
|  |  | Perceived racist treatment by health professionals | “*Although you speak their language. I am an African, I haven’t been in this country too long, but I am trying very hard to talk slowly and clearly... Sometimes, they probably think that if you are black, you wouldn’t understand anyway*” [P] [36] |

^1^ [P] denotes a participant comment; [A] denotes an author comment.
